# Long non-coding RNA TUG1 aggravates cerebral ischemia and reperfusion injury by sponging miR-493-3p/miR-410-3p

**DOI:** 10.1515/med-2021-0253

**Published:** 2021-06-24

**Authors:** Jinlong Du, Wenjing Li, Bing Wang

**Affiliations:** Department of Critical Care Medicine, Jingzhou Central Hospital, The Second Clinical Medical College of Yangtze University, Jingzhou, Hubei, 434020, China; Department of Ultrasound, Jingzhou Central Hospital, The Second Clinical Medical College of Yangtze University, Jingzhou, Hubei, 434020, China

**Keywords:** cerebral ischemic reperfusion injury, TUG1, miR-493-3p, miR-410-3p, p-JNK and p38 pathways

## Abstract

**Background:**

Cerebral ischemia and reperfusion injury (CIRI) affects bodily function by causing irreversible damage to brain cells. The diverse pathophysiological course factors hinder the research work to go deeper. Long noncoding RNA taurine-upregulated gene 1 (TUG1) has been reported to be related to CIRI. This study explored the undefined regulatory pathway of TUG1 in CIRI.

**Methods:**

Quantitative real-time polymerase chain reaction was applied to test the expression of TUG1, microRNA (miR)-493-3p and miR-410-3p. The viability and apoptosis of oxygen and glucose deprivation/reoxygen (OGD/R) model cells were evaluated by cell counting kit-8 and flow cytometry assay, respectively. The determination of inflammatory factors of interleukin-6, interleukin-1β and tumor necrosis factor-α was presented by enzyme-linked immunosorbent assay. The oxidative stress was performed by measuring the generation of malondialdehyde, reactive oxygen species and the activity of superoxide dismutase. Cytotoxicity was presented by measuring the generation of lactate dehydrogenase. Western blot assay was devoted to assessing the level of apoptosis-related factors (cleaved-caspase-3 and cleaved-caspase-9) and the protein level of c-Jun N-terminal kinase (JNK) and p38 mitogen-activated protein kinase (p38 MAPK) pathway-related factors in neuro-2a cells treated by OGD/R. Besides, online database starBase was applied to predict the potential binding sites of TUG1 to miR-493-3p and miR-410-3p, which was further confirmed by the dual-luciferase reporter system.

**Results:**

The expression of TUG1 was upregulated, while miR-493-3p or miR-410-3p was downregulated in the serum of CIRI and OGD/R model cells. Meanwhile, knockdown of TUG1 eliminated the suppression in proliferation, the promotion in apoptosis, inflammation and oxidative stress, as well as the cytotoxicity in OGD/R model cells. Interestingly, the inhibition of miR-493-3p or miR-410-3p allayed the above effects. In addition, TUG1 harbored miR-493-3p or miR-410-3p and negatively regulated their expression. Finally, the TUG1 activated JNK and p38 MAPK pathways by sponging miR-493-3p/miR-410-3p.

**Conclusion:**

TUG1 motivated the development of CIRI by sponging miR-493-3p/miR-410-3p to activate JNK and p38 pathways. The novel role of TUG1 in CIRI may contribute to the advancement of CIRI treatment.

## 1 Introduction

Cerebral ischemia and reperfusion injury (CIRI) causes irreversible brain damage. Annually, out of 15 million people unfortunately afflicted with CIRI, about a third of them died and more than a third suffered permanent disability [1]. The surviving CIRI patients brought continuous pain and suffering to themselves and their families due to the drastic changes of emotion and behavior [2]. Due to the extreme sensitivity of cerebrum to oxygen and blood, even a few minutes of deprivation could cause permanent injury to the brain [3,4]. In the process of ischemia, the production of adenosine triphosphate (ATP) and intracellular pH (pondus hydrogenii) level were reduced. The excess hydrogen ions were pumped out of cells by the exchange of Na^+^/H^+^; however, this led to the accumulation of sodium ions in the cell and the disorder of ATPase-dependent transport mechanism, which induced intracellular calcium overload [5]. Following this, the accumulation of intracellular calcium resulted in the release of apoptosis-inducing factors (e.g., caspase-3, caspase-9) by cell or mitochondrial breakdown [6]. After reperfusion, the production of oxidative stress products, including reactive oxygen species (ROS), superoxide dismutase (SOD) and malondialdehyde (MDA), lactate dehydrogenase (LDH), increased sharply, and the proinflammatory neutrophils (e.g., interleukin-6 [IL-6], interleukin-1β [IL-1β] and tumor necrosis factor-α [TNF-α]) aggravated the ischemic injury by infiltrating into the ischemic tissues [7,8]. However, the detailed pathogenesis and progression have not been clearly studied. This study is concentrated on exploring the regulatory pathway of CIRI, so as to provide a novel theoretical basis for the treatment of CIRI.

During the study of the occurrence and development of CIRI, long non-coding RNAs (lncRNAs) came into the field of vision attributed to the vital role in CIRI process. lncRNAs contain more than 200 nucleotides and are highly correlated with genetic imprinting [9]. Numerous research studies have explored the interaction between lncRNAs and CIRI. lncRNA small nuclear RNA host gene 16 overexpression dispelled CIRI progression by harboring microRNA (miR)-106b-5p to regulate LIM domain kinase 1 [10]. lncRNA metastasis-associated lung adenocarcinoma transcript 1 knockdown mitigated CIRI through miR-145/aquaporin 4 (AQP4) pathway [11]. lncRNA CAMK2D-associated transcript 1 protected against CIRI by Ca2+/calmodulin-dependent protein kinase II-δ/nuclear factor kappa-B signal pathway [12]. These studies highlighted the crucial role of lncRNAs that might serve as a biomarker in CIRI. lncRNA taurine-upregulated gene 1 (TUG1) has been reported to be dysregulated in various diseases, including CIRI. Shen et al. demonstrated that TUG1 inhibited the metastasis of colorectal cancer cells by regulating transforming growth factor beta/twist family bHLH transcription factor 1/epithelial–mesenchymal transition pathway [13]. The abnormal upregulation of TUG1 induced the apoptosis via downregulating Krüppel-like factor 2 in hepatocellular carcinoma [14]. TUG1 was highly expressed, which expedited CIRI by miR-145/AQP4 pathway [15]. However, the specific pathway of TUG1 to participate in the regulation of CIRI remains to be further studied.

The regulatory mechanism of lncRNA on gene expression at transcription and translation levels has been widely researched. lncRNAs could act as an endogenous sponge for microRNAs (miRNAs) to suppress miRNA functions in cells [16]. For example, lncRNA TUG1 regulates microglial polarization after oxygen–glucose deprivation by sponging miR-145a-5p [17]. Besides, TUG1 could promote colon cancer progression via regulating miR-26a-5p/matrix metalloproteinases-14/p38 mitogen-activated protein kinase (p38 MAPK)/heat shock protein 27 pathway [18]. Numerous studies have shown that MAPK/p38 pathway is activated by various inflammatory extracellular mediators, while c-Jun N-terminal kinases (JNK) isoforms are strongly activated during various cellular stress responses [19]. Furthermore, JNK and p38 MAPK signaling pathways were reported to be involved in regulating the progression of CIRI [20]. Liu et al. uncovered that the activation of JNK and p38 pathways was inhibited, while the ERK1/2 was activated by electroacupuncture treatment in CIRI rats [21]. Furthermore, all-trans retinoic acid could inhibit the JNK/p38 MAPK pathway to alleviate blood–brain barrier disruption, thereby ameliorating the early experimental CIRI in rats [22]. Thus, we wondered if TUG1 exerted its function in CIRI by sponging miRNAs to regulate the activity of JNK/p38 MAPK pathway.

In this study, the dysregulation of TUG1 was found to be interacted with the progression of CIRI. Moreover, TUG1 knockdown-induced repression in apoptosis, inflammation and oxidative stress, cytotoxicity and promotion in proliferation, suppression in activating JNK and p38 MAPK pathways could be allayed by the inhibition of miR-493-3p/miR-410-3p in oxygen and glucose deprivation/reoxygen (OGD/R) model cells. This study may present a potential target site for CIRI therapy.

## 2 Materials and methods

### 2.1 Collection of human peripheral blood samples

The ethics committee of Jingzhou Central Hospital, The Second Clinical Medical College of Yangtze University accredited this research. All participants or their statutory guardians signed the informed consent for this study. A total of 25 healthy volunteers’ and 25 CIRI patients’ peripheral blood samples (5 mL per person) were collected in pre-labeled test tubes from Jingzhou Central Hospital, The Second Clinical Medical College of Yangtze University. To isolate the serum that was used to measure the subsequent indicators, centrifugation (5,000 rpm, 15 min, 4°C) was performed on the blood samples.

### 2.2 Cell culture and the establishment of OGD/R model

Neuro-2a (N2a) cell line (American Type Culture Collection, Manassas, VA, USA), mouse neuroblastoma cells, was adopted to perform this research. DMEM (Gibco, Carlsbad, CA, USA) with 10% fetal bovine serum (PAN, Bavaria, Germany) and Penicillin/Streptomycin was devoted to culturing cells in an incubator with 5% CO_2_ at 37°C. For the establishment of OGR/D model, the medium without glucose and serum and an incubator with 1% O_2_, 5% CO_2_ and 94% N_2_ were applied to stimulate the ischemic condition. The complete DMEM (Gibco) with normoxic environment was employed to mimic reoxygenation. Briefly, N2a cells were cultured in the ischemic condition for 6, 12, 24 h, following incubation in reoxygenation condition for another 24 h at 37°C. For the control group, N2a cells were cultured in normal medium with 95% air, 5% CO_2_ at 37°C.

### 2.3 Cell transfection

For the gain- and loss-of-function approaches, N2a cells were transfected with small interfering RNA stable TUG1 knockdown (si-TUG1^#1^, si-TUG1^#2^), antisense RNA (anti-RNA) against miR-493-3p (anti-miR-493-3p) or miR-410-3p (anti-miR-410-3p) or the negative controls (si-NC, miR-NC, anti-miR-NC). All the transfection sequences and carriers were provided by GenePharma (Shanghai, China).

### 2.4 RNA extraction and real-time quantitative polymerase chain reaction (RT-qPCR)

TRIzol reagent (Thermo Fisher Scientific, Waltham, MA, USA) was used to extract the total RNA from N2a cells. Following this, the DU800 UV/Vis spectrophotometer (Beckman Coulter, CA, USA) was used to detect the purity of the extracted RNA. The reverse transcription was performed using cDNA reverse transcription kit (Applied Biosystems, Foster City, CA, USA). The next sequence amplification was performed using Prime Script™ RT reagent kit (Takara, Shiga, Japan). SYBR Green Master Mix kit (Takara) together with ABI7500 real-time PCR system (Thermo Fisher Scientific) were used for RT-qPCR. Glyceraldehyde 3-phosphate dehydrogenase (GAPDH) and U6 small nuclear RNA (U6) were responsible for the normalization of RNA expression. The quantitative analysis was performed by the 2^−ΔΔCt^ method. The relevant primer sequences were presented as follows: TUG1 forward, 5′-GTGAAGGTCACTGGACCCTG-3′, reverse, 5′-CGGTCACAAAATGCATAGAGGT-3′; miR-493-3p, forward, 5′-GCGCGTGAAGGTCTACTGTGT-3′, reverse, 5′-AGTGCAGGGTCCGAGGTATT-3′; miR-410-3p, forward, 5′-CGCGCGAATATAACACAGATG-3′, reverse, 5′-GAGAACAGCTCTGTGTTATAT-3′; GAPDH, forward, 5′-CAGTCAGCCGCATCTTCTTTT-3′, reverse, 5′-GTGACCAGGCGCCCAATAC-3′; U6 forward, 5′-GCTTCGGCAGCACATATACTAAAAT-3′, reverse, 5′-CGCTTCACGAATTTGCGTGTCAT-3′.

### 2.5 Cell viability

Cell counting kit-8 assay (CCK-8; Hanbio Biotechnology Co., Ltd., Shanghai, China) was devoted to assessing the viability of N2a cells treated by OGD/R. Briefly, OGD/R model cells were cultured in 96-well plates with a cell density of 2 × 10^3^ per well for 24 h. In order to visualize the absorbance at 450 nm, the cells were first stained by CCK-8 reagent. Then, the result was measured by MultiMode Microplate Reader (Thermo Fisher Scientific).

### 2.6 Cell apoptosis

Cells were incubated with OGD/R after transfection. Then, cell apoptosis was evaluated by Annexin V-Fluorescein isothiocyanate/propidium iodide staining kit (Biouniquer Technology, Nanjing, China) together with BD FACS Canto™ II flow cytometer (BD Biosciences, San Jose, CA, USA) in N2a cells treated by OGD/R according to the manufacturer’s instruction manual.

### 2.7 Western blot

Western blot assay was performed according to previous protocols [11]. The protein expression of cleaved-caspase-3 (cleaved-cas-3) and cleaved-caspase-9 (cleaved-cas-9), phosphorylation-c-Jun N-terminal kinase (p-JNK) and JNK, phosphorylation-p38 the mitogen-activated protein kinase (p-p38), p38, GADPH was measured by western blot in N2a cells. First, N2a cells were lysed by RIPA buffer (Beyotime). Then, the proteins were isolated by sodium dodecyl sulfate polyacrylamide gel electrophoresis (Beyotime) and polyvinylidene difluoride membrane (Millipore, Billerica, MA, USA). Afterward, the primary antibodies were incubated with the blocked membrane for 12 h at 4°C. In order to eliminate the unwanted distractions, 1× tris-buffered saline tween-20 (TBST) was applied to wash away the excess residues. The secondary antibody was incubated with the complexes for 1 h at room temperature. Eventually, the interested protein expressions were measured by Electrochemiluminescence detection kit (Roche Diagnostics GmbH, Mannheim, Germany). The antibodies were cleaved-cas-3, cleaved-cas-9, p-JNK and JNK, p-p38, p38 (1:1,000; Cell Signaling, Danvers, MA, USA), GAPDH (1:500; Santa Cruz, Dallas, TX, USA) and HRP-conjugated secondary antibodies (1:5,000; Southern-Biotech, Birmingham, AL, USA).

### 2.8 Enzyme-linked immunosorbent assay (ELISA)

The production of IL-6, IL-1β and TNF-α in N2a cells treated by OGD/R was measured by ELISA kits (Beyotime). Briefly, the cell culture medium supernatants were collected to perform the ELISA following the manufacturer’s instruction. A microplate reader was devoted to detecting the absorbance at 450 nm. The production of TNF-α, IL-6 and IL-1β was normalized by the standard curve made in advance by using the standard solution.

### 2.9 Determination of cellular ROS

Reactive Oxygen Species Assay kit (Beyotime Institute of Biotechnology, Haimen, China) was used to detect the intercellular production of ROS following the protocol. Cells were lysed and centrifuged (3,000 rpm, 15 min, 4°C) to separate the supernatant for performing the determination of ROS production. A flow cytometry (Thermo Fisher scientific) was used to measure the generation of ROS in N2a induced by OGD/R.

### 2.10 Assessment of MDA, SOD production

The production of MDA and the activity of SOD were measured by colorimetry. Briefly, cells were lysed and centrifuged (3,000 rpm, 15 min, 4°C) to separate the supernatant. Then, the production of ROS was assessed by Reactive oxygen species assay kit (Solarbio Science & Technology Co., Ltd., Beijing, China). The production of MDA and the activity of SOD were measured by a spectrometer (Clontech, Mountain View, CA, USA) and normalized by the standard curve made in advance by using the standard solution.

### 2.11 Evaluation of LDH release

Cytotoxicity can be determined by measuring the level of LDH in cell supernatant by colorimetry. N2a cells were first treated with OGD/R after transfection. The cells were then collected and centrifuged (3,000 rpm, 15 min, 4°C) to separate the supernatant. Then, Cytotoxicity LDH Assay Kit (Dojindo, Kumamoto, Japan) was used to determine the level of LDH. The production of LDH was normalized by the standard curve made in advance by using the standard solution.

### 2.12 Dual-luciferase reporter assay

The underlying interaction between TUG1 and miR-493-3p or miR-410-3p was predicted by online database starBase. Dual-Luciferase miRNA Target Expression Vector pmirGLO vectors (Promega, Madison, WI, USA) were applied to confirm the prediction. The putative combinational sequences or the mutant sequences without the combinational sites between TUG1 and miR-493-3p or miR-410-3p were cloned into pmirGLO vectors, named as TUG1-WT or TUG1-MUT. Then, the constructed vectors together with miR-493-3p or miR-410-3p were co-transfected into N2a cells by Lipofectamine 2000 Transfection Reagent (Invitrogen, Waltham, MA, USA). Dual-Luciferase Reporter Assay (Promega) was applied to detect the activity of luciferase. Renilla luciferase activity was used as the normalization standard.

### 2.13 Statistical analysis

GraphPad Prism version 7.0 (GraphPad Inc., San Diego, CA, USA) was applied to analyze the data. Each experiment was repeated at least three times and presented as mean ± SD. *P* < 0.05 held up as a standard for judging the statistical significance. The difference between two groups was analyzed by Student’s *t*-test, while among multiple groups was analyzed by one-way analysis of variance.

## 3 Results

### 3.1 Upregulation of TUG1 was associated with CIRI

**Figure 1:**
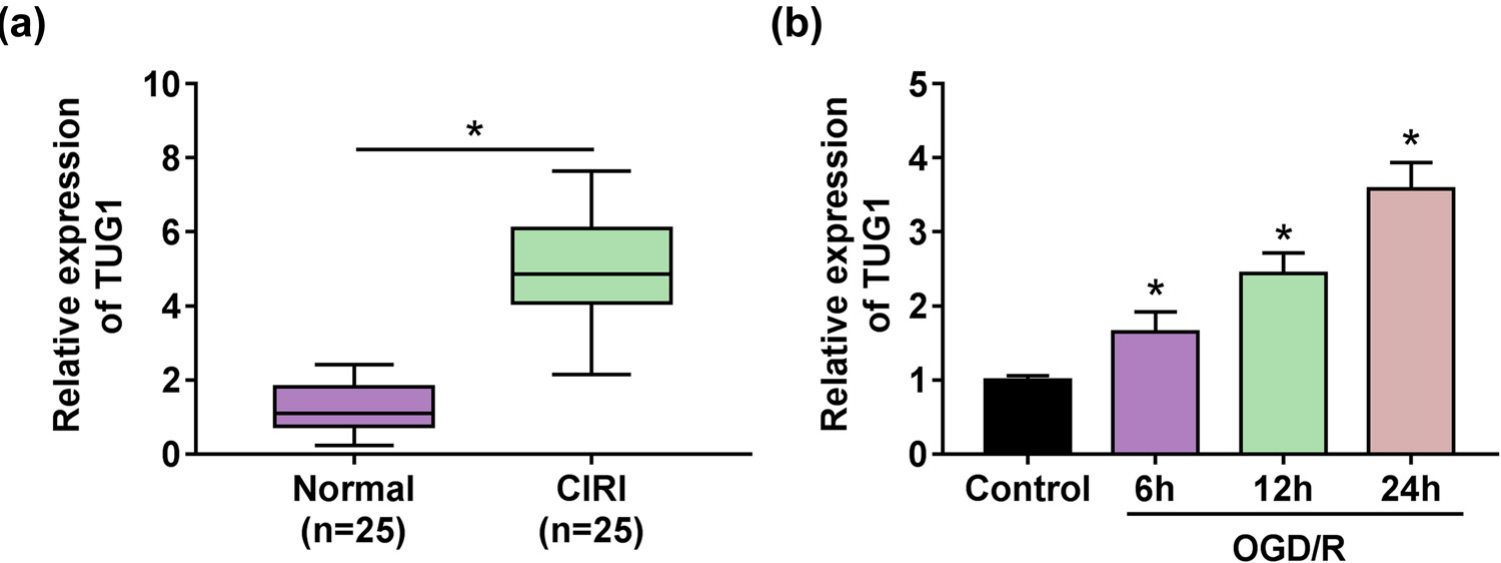
TUG1 was involved in CIRI. (a) The expression of TUG1 was measured by RT-qPCR in the serum of CIRI patients (*n* = 25) and normal people (*n* = 25). (b) RT-qPCR was devoted to testing the expression of TUG1 in N2a cells with or without OGD/R treatment when the cultivation time reaches 6, 12 and 24 h. *P < 0.05.

Given the close relationship between the dysregulation of TUG1 and the development of diseases, the expression of TUG1 in the serum of CIRI patients and normal subject was detected. A striking increase of TUG1 expression in the serum of CIRI patients (Figure 1a) inspired us to build a cellular model of CIRI by OGD/R. As expected, the expression of TUG1 was significantly increased with the prolongation of the treatment time (Figure 1b). These data imply the participatory role of TUG1 in CIRI.

### 3.2 TUG1 knockdown promoted cell viability and suppressed apoptosis in OGD/R model cells

**Figure 2:**
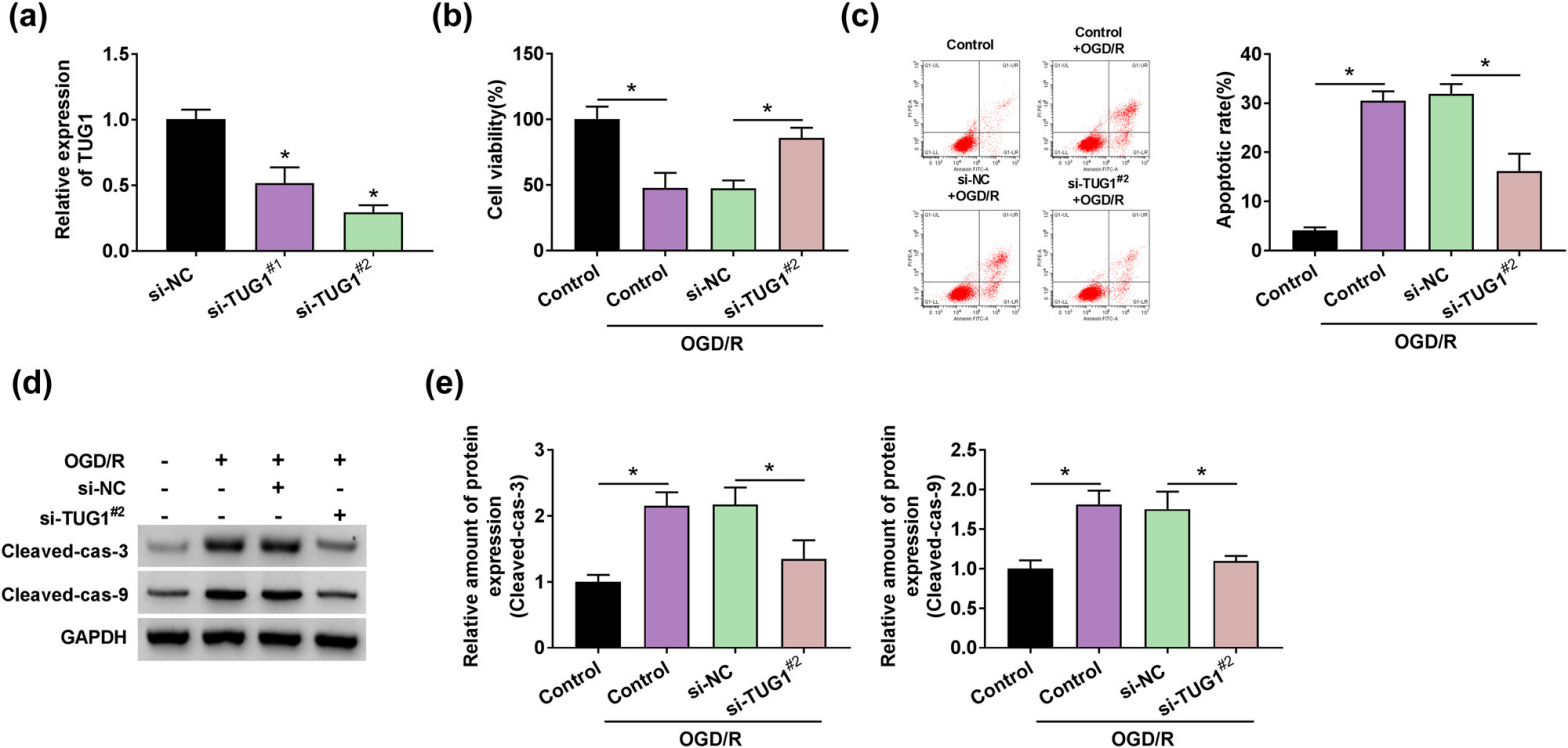
Knockdown of TUG1 hindered OGD/R-mediated decrease in viability and increase in apoptosis in N2a cells *in vitro*. (a) The transfection efficiency of si-TUG1 (si-TUG1^#1^, si-TUG1^#2^) was assessed by RT-qPCR in N2a cells. (b) The effect of si-TUG1^#2^ on cell viability was evaluated by CCK-8 assay in N2a cells treated by OGD/R. (c) Flow cytometry assay was applied to detect the apoptosis rate in N2a cells treated by OGD/R. (d and e) The proteins levels of apoptosis-related markers (cleaved-cas-3 and cleaved-cas-9) were performed by western blot assay in N2a cells treated by OGD/R. *P < 0.05.

Given the participatory role of TUG1 in CIRI, we wondered whether TUG1 affected the biological behaviors of CIRI cells. Loss-of-function experiments on TUG1 were performed in OGD/R model cells. Due to the excellent suppression performance in OGD/R model cells, si-TUG1^#2^ was chosen as a presider in follow-up experiments (Figure 2a). Interestingly, the absence of TUG1 represented a promotion in cell viability, which reduced by OGD/R treatment in N2a cells (Figure 2b). The promotion of apoptosis rate, which induced by OGD/R treatment, was inhibited by si-TUG1^#2^ in N2a cells (Figure 2c). This finding was supported by the result of protein expression of apoptosis-related markers (cleaved-cas-3 and cleaved-cas-9) (Figure 2d and e). These data indicated that TUG1 functionally affected the proliferation and apoptosis of CIRI cells.

### 3.3 TUG1 knockdown repressed the inflammation, oxidative stress and cytotoxicity in OGD/R model cells

**Figure 3:**
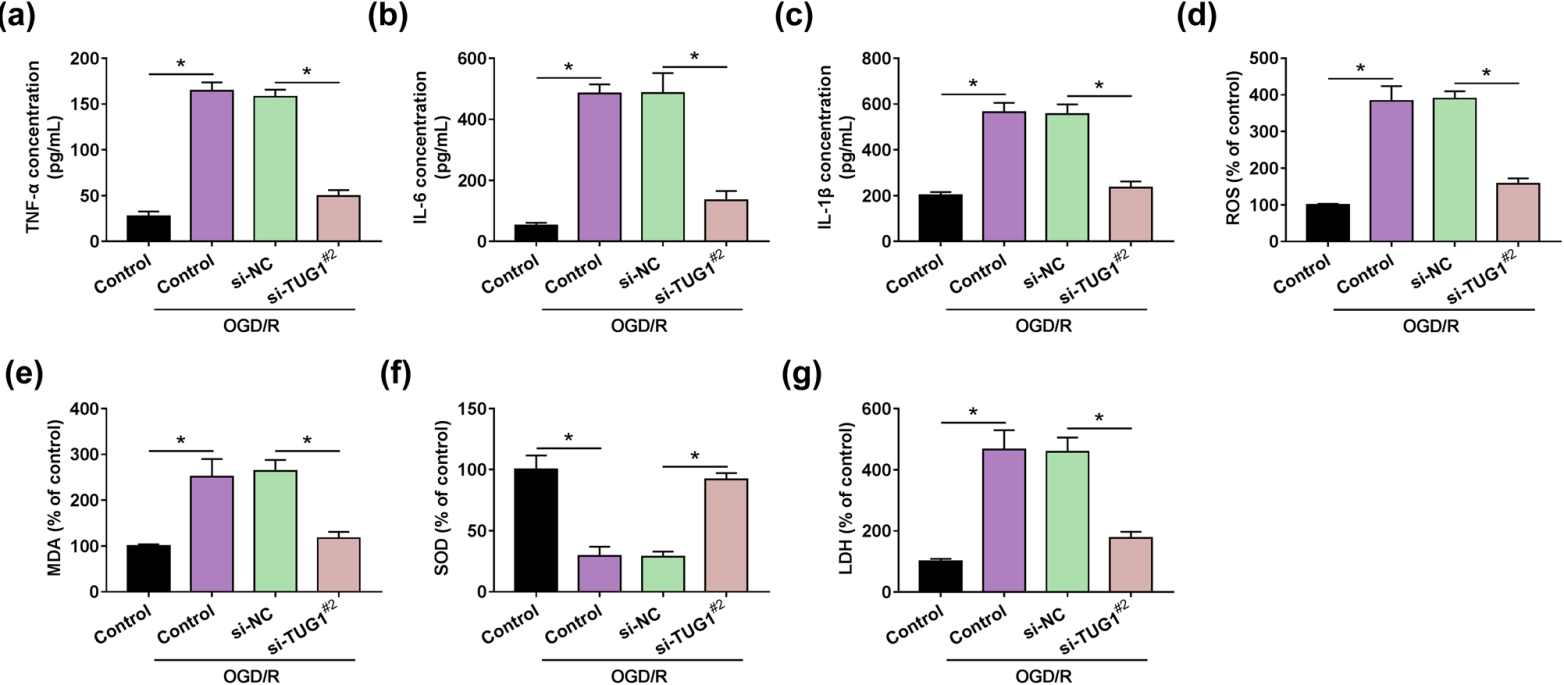
Knockdown of TUG1 attenuated the effects of OGD/R on the inflammation and oxidative stress of N2a cell. (a–c) The concentration of TNF-α , IL-6 and IL-1β was measured by ELISA in N2a cells with or without being treated by OGD/R. (d) Reactive oxygen species assay kit analyzed the ROS generation in N2a cells with or without being treated by OGD/R. (e–g) The intracellular levels of MDA, SOD and LDH were measured by colorimetry. *P < 0.05.

Given the functional effects of TUG1, we further explored the role of TUG1 in inflammation and oxidative stress accompanied with CIRI. TNF-α , IL-6 and IL-1β were the representative markers of cell inflammation. The concentration of inflammation-related markers (TNF-α , IL-6 and IL-1β) showed a significant increase, which was induced by OGD/R treatment, and si-TUG1^#2^ leads to a strong reduction in this elevation in OGD/R model cells (Figure 3a–c). ROS, MDA and SOD were the typical indicators of cellular oxidative stress, and LDH symbolized the cytotoxicity. Likewise, si-TUG1^#2^ provided a reversion on the promotion of the production of ROS, MDA and the inhibition of SOD activity mediated by OGD/R treatment in N2a cells (Figure 3d–f). Moreover, the production of cytotoxicity-related marker LDH showed that si-TUG1^#2^ allayed the cytotoxicity mediated by OGD/R treatment in N2a cells (Figure 3g). These data indicated that TUG1 knockdown alleviated the damage caused by OGD/R in N2a cells.

### 3.4 TUG1 acted as a molecular sponge and negatively regulated the expression of miR-493-3p/miR-410-3p

**Figure 4:**
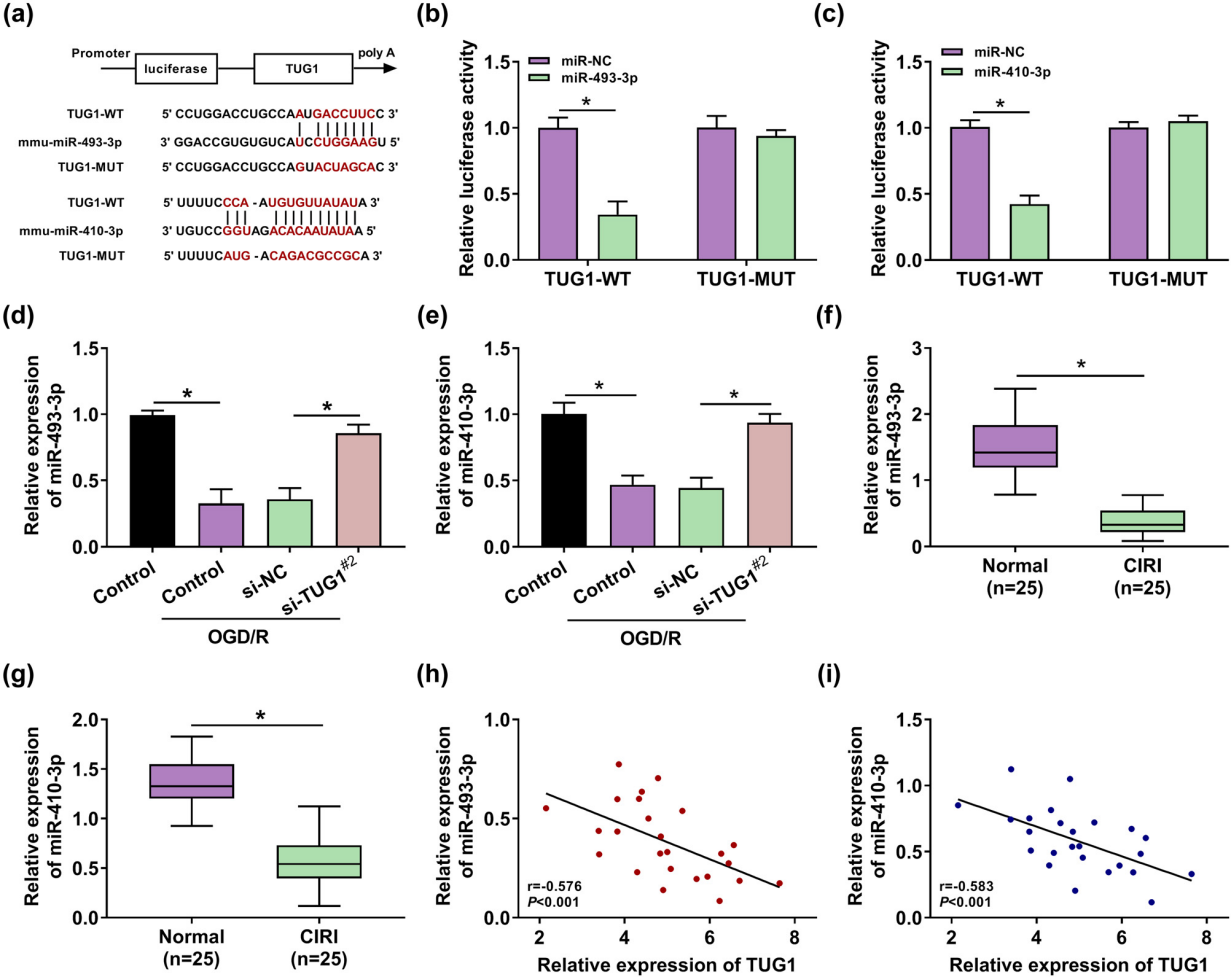
lncRNA TUG1 negatively regulated the expression of miR-493-3p and miR-410-3p by sponging miR-493-3p and miR-410-3p. (a) Online database starBase was adopted to make inferences about the underlying binding sequences between TUG1 and miR-493-3p or miR-410-3p in N2a cells. (b and c) Dual-luciferase reporter system presented the combination between TUG1 and miR-493-3p or miR-410-3p in N2a cells. (d and e) The expression of miR-493-3p and miR-410-3p was measured by RT-qPCR in N2a cells with or without being treated by OGD/R. (f and g) The expression of miR-493-3p and miR-410-3p was measured by RT-qPCR in the serum of CIRI patients and normal people. (h and i) Spearman’s correlation analysis was employed to analyze the relationship between the expression of TUG1 and miR-493-3p or miR-410-3p. *P < 0.05.

Given the obstructive role of si-TUG1 in CIRI, the deeper regulatory mechanism of TUG1 that participated in CIRI progression has caught our attention. The potential binding sites between TUG1 and miRNAs were predicted by online database starBase. Thus, miR-493-3p and miR-410-3p came into our view due to the prediction of existing binding sequences between TUG1 and miR-493-3p or miR-410-3p (Figure 4a). Furthermore, the interaction between TUG1 and miR-493-3p or miR-410-3p was verified by the decrease in luciferase in TUG1-WT (Figure 4b and c). The expression of miR-493-3p or miR-410-3p was inhibited in OGD/R model cells and the serum of CIRI patients, which presented a strong connection between CIRI and miR-493-3p or miR-410-3p (Figure 4d–g). Interestingly, si-TUG1^#2^ could relieve the inhibition of the expression of miR-493-3p or miR-410-3p induced by OGD/R treatment in N2a cells (Figure 4d and e). Meanwhile, Spearman’s correlation analysis indicated a negative correlation between the expression of TUG1 and miR-493-3p or miR-410-3p (Figure 4h and i). These data meant that TUG1 adsorbs miR-493-3p or miR-410-3p to participate in CIRI progression.

### 3.5 TUG1 sponged miR-493-3p or miR-410-3p to regulate the proliferation and apoptosis in OGD/R model cells

**Figure 5:**
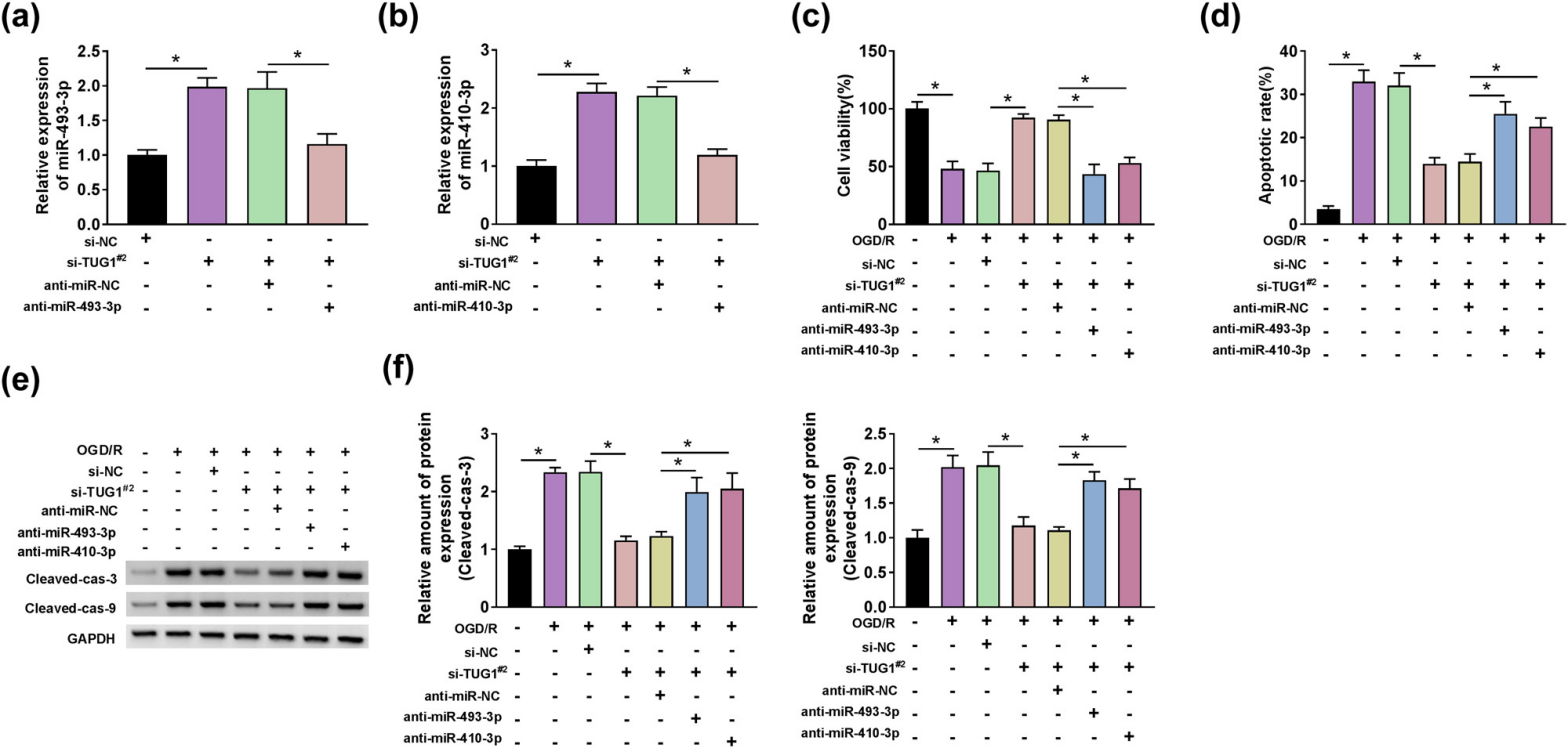
The inhibition of miR-493-3p or miR-410-3p could restore the reverse effects of TUG1 knockdown on OGD/R-mediated decrease in viability and increase in apoptosis in N2a cells *in vitro*. (a and b) The effect of si-TUG1^#2^ and anti-miR-493-3p or anti-miR-410-3p on the expression of miR-493-3p or miR-410-3p was detected by RT-qPCR. (c) The effect of si-TUG1^#2^ and anti-miR-493-3p or anti-miR-410-3p on cell viability was evaluated by CCK-8 assay in N2a cells treated by OGD/R. (d) Flow cytometry assay was applied to detect the apoptosis rate in N2a cells, which transfected with si-TUG1^#2^ + anti-miR-493-3p or si-TUG1^#2^ + anti-miR-410-3p or negative controls, with or without being treated by OGD/R. (e and f) The proteins levels of apoptosis-related markers (cleaved-cas-3 and cleaved-cas-9) were performed by western blot assay in N2a cells, which transfected with si-TUG1^#2^ + anti-miR-493-3p or si-TUG1^#2^ + anti-miR-410-3p or negative controls, with or without being treated by OGD/R. *P < 0.05.

Given the mechanical relationship between TUG1 and miR-493-3p or miR-410-3p, we deeply studied the functional interaction between TUG1 and miR-493-3p or miR-410-3p. First, si-TUG1^#2^ leads to a significant increase in the expression of miR-493-3p or miR-410-3p, which was restored by anti-miR-493-3p or anti-miR-410-3p, respectively, in N2a cells (Figure 5a and b). Moreover, anti-miR-493-3p or anti-miR-410-3p acted as a restorer in the promotion of viability induced by si-TUG1^#2^ in OGD/R model cells (Figure 5c). Differentially, si-TUG1^#2^-mediated inhibition in apoptosis could be ameliorated by anti-miR-493-3p or anti-miR-410-3p in OGD/R model cells (Figure 5d–f). These data declared that the proliferation and apoptosis could be regulated by TUG1, which interacted with miR-493-3p or miR-410-3p in OGD/R model cells.

### 3.6 TUG1 regulated inflammation, oxidative stress and cytotoxicity by sponging miR-493-3p or miR-410-3p in OGD/R model cells

**Figure 6:**
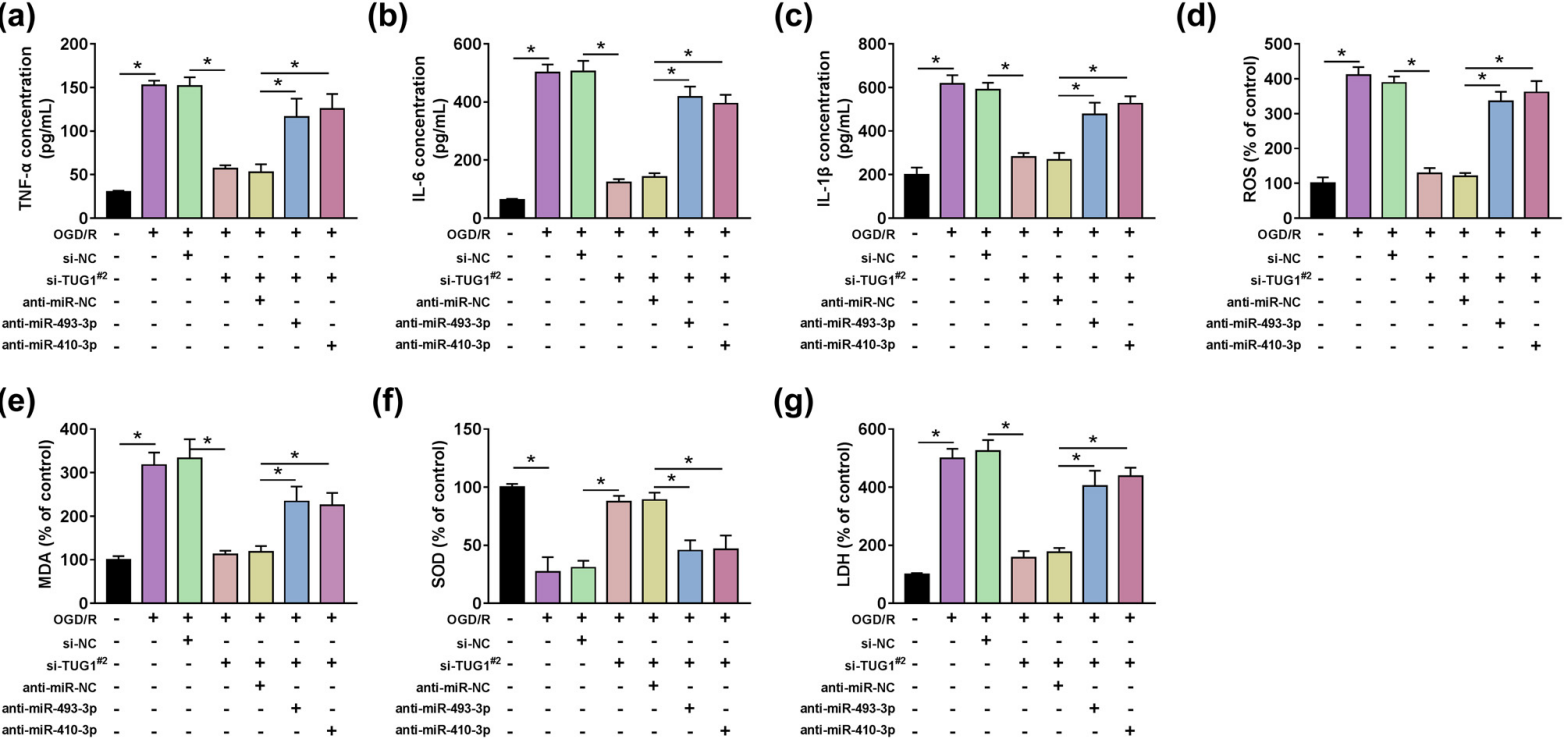
The inhibition of miR-493-3p or miR-410-3p could restore the reverse effects of TUG1 knockdown on OGD/R-mediated decrease in inflammation and oxidative stress in N2a cells *in vitro*. (a–c) The concentration of TNF-α , IL-6 and IL-1β was measured by ELISA in N2a cells, which transfected with si-TUG1^#2^ + anti-miR-493-3p or si-TUG1^#2^ + anti-miR-410-3p or negative controls, with or without being treated by OGD/R. (d) The effect of si-TUG1^#2^ and anti-miR-493-3p or anti-miR-410-3p on ROS generation was analyzed by Reactive oxygen species assay kit in N2a cells with or without being treated by OGD/R. (e–g) The intracellular levels of MDA, SOD and LDH were measured by colorimetry in N2a cells, which transfected with si-TUG1^#2^ + anti-miR-493-3p or si-TUG1^#2^ + anti-miR-410-3p or negative controls, with or without being treated by OGD/R. *P < 0.05.

Given the regulation relationship between TUG1 and miR-493-3p or miR-410-3p, we paid attention to the functional relationship between TUG1 and miR-493-3p or miR-410-3p in inflammation and oxidative stress in OGD/R model cells. The concentration of TNF-α , IL-6 and IL-1β delivered the clear message that si-TUG1^#2^ showed a suppression effect on inflammation, which was counteracted by anti-miR-493-3p or anti-miR-410-3p in OGD/R model cells (Figure 6a–c). The production of ROS, MDA and SOD, LDH illustrated that anti-miR-493-3p or anti-miR-410-3p could mitigate the suppression in oxidative stress and cytotoxicity mediated by si-TUG1^#2^ in OGD/R model cells (Figure 6d–g). These data manifested TUG1 sponged miR-493-3p or miR-410-3p to regulate the inflammation, oxidative stress and cytotoxicity in OGD/R model cells.

### 3.7 TUG1 sponged miR-493-3p or miR-410-3p to activate JNK and p38 pathways

**Figure 7:**
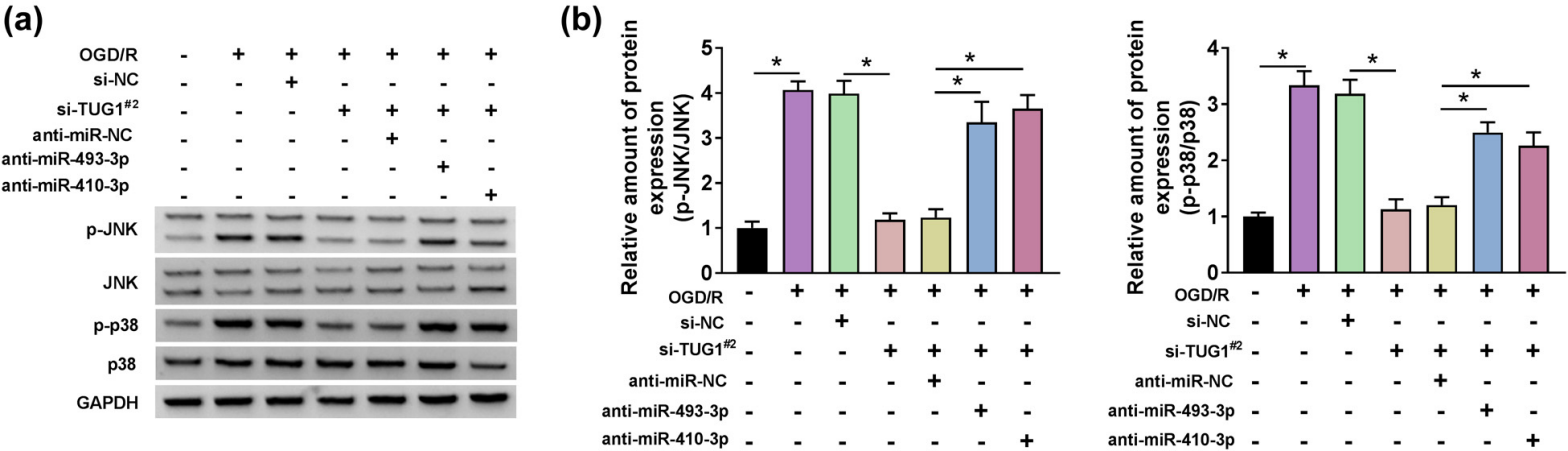
The inhibition of miR-493-3p or miR-410-3p could restore the reverse effects of TUG1 knockdown on OGD/R-mediated activation in p-JNK/JNK and p-p38/p38 pathway in N2a cells *in vitro*. (a and b) The protein expression of p-JNK, JNK and p-p38, p38 was measured by western blot in N2a cells, which transfected with si-TUG1^#2^ + anti-miR-493-3p or si-TUG1^#2^ + anti-miR-410-3p or negative controls, with or without being treated by OGD/R. *P < 0.05.

Given the functional relationship between TUG1 and miR-493-3p or miR-410-3p in inflammation, oxidative stress and cytotoxicity, we further explored the downstream signaling pathways. As signal pathways closely related to inflammation and apoptosis in MAPK, the changes of p-JNK/JNK and p-p38/p38 were also captured in OGD/R model cells. Intuitively, the relative ratio of p-JNK/JNK and p-p38/p38 was improved by the treatment of OGD/R in N2a cells. Meanwhile, anti-miR-493-3p or anti-miR-410-3p dispelled the restraint induced by si-TUG1^#2^ in the relative ratio of p-JNK/JNK and p-p38/p38 in OGD/R model cells (Figure 7a and b). These data uncovered that TUG1 activated JNK and p38 pathways by harboring miR-493-3p or miR-410-3p.

### 3.8 TUG1 participated in the regulatory network of CIRI process

**Figure 8:**
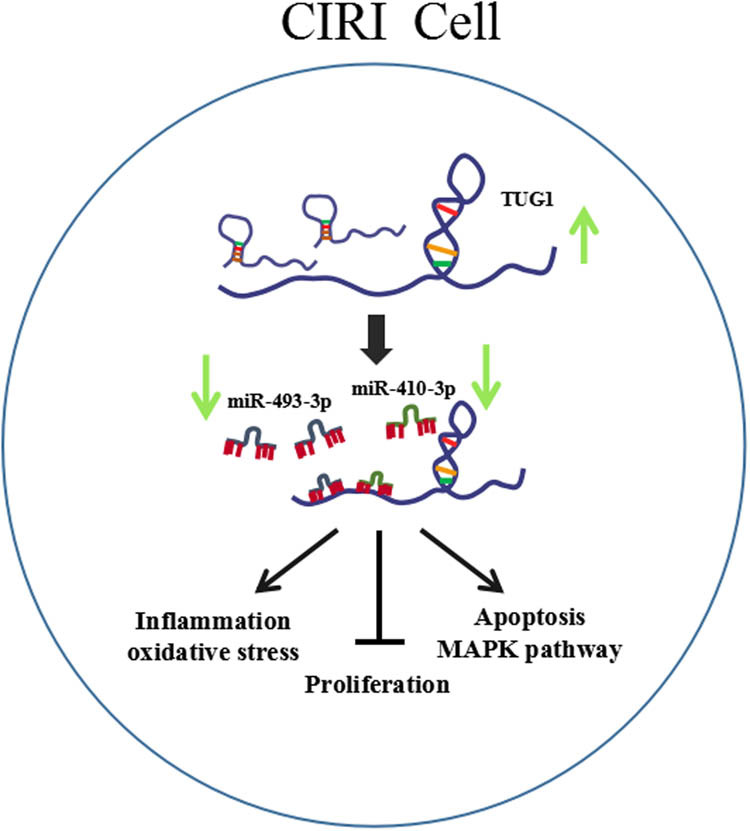
Schematic diagram of TUG1 involved in CIRI regulation. TUG1 was highly expressed in the serum of CIRI patients. TUG1 inhibited the proliferation, promoted inflammation, oxidative stress and apoptosis, cytotoxicity and activated JNK and p38 pathways by sponging miR-493-3p or miR-410-3p in CIRI progression.

Overall, a schematic diagram (Figure 8) was presented to better understand the regulatory mechanism of TUG1 in CIRI progress. TUG1 was upregulated in the serum of CIRI patients. As a molecular sponge, TUG1 negatively regulated the expression of miR-493-3p or miR-410-3p to inhibit the proliferation, promote inflammation, oxidative stress and apoptosis, cytotoxicity and activate MAPK pathway in CIRI progression.

## 4 Discussion

The multifactorial pathogenic mechanisms assisted CIRI in evading the precise treatment. The main pathogenic mechanisms of CIRI are listed as follows: (1) calcium overload, (2) oxidative/nitrosative stress, (3) endoplasmic reticulum stress, (4) mitochondrial dysfunction, (5) activation of apoptotic and autophagic pathways, (6) protein kinases, (7) epigenetic changes, (8) inflammation, (9) protein cleavage products and other degradation products, (10) no-reflow and (11) genomic/metabolomic insights [7]. According to the above mechanisms, a number of studies have been conducted on CIRI [23–29]. The interaction between lncRNAs and CIRI has been widely studied. Gai et al. illustrated that the upregulation of CHRF boosted the progression of CIRI [30]. Liang et al. proposed that the apoptosis and inflammation were promoted by MEG3 in CIRI [31]. Accordingly, the information about the motivational role of lncRNAs may provide a therapeutic strategy for CIRI. Attention to this study was focused on the functional role of TUG1 in apoptosis, inflammation, oxidative stress and protein kinases, which were accompanied by CIRI. Primarily, the abnormal increase in the expression of TUG1 in the serum of CIRI patients and OGD/R model cells was further verified by the facilitator role of TUG1 in CIRI [15]. In our research, TUG1 expression was upregulated in the serum of CIRI patients, as well as in OGD/R model cells. TUG1 knockdown resulted in a promotion in proliferation and an inhibition in apoptosis, inflammation and oxidative stress, cytotoxicity. TUG1 knockdown inhibited cell proliferation but promoted cell apoptosis in OGD/R model cells. Taken together, these data highlighted the promotion effect of TUG1 on CIRI progression.

lncRNAs were regularly recognized as competing endogenous RNAs to associate with disease progression by sponging miRNAs [32–36]. The upregulation of TUG1 leads to a suppression in proliferation and a promotion in apoptosis, which was reversed by miR-142-3p in myocardial injury during ischemia and reperfusion [37]. TUG1 was highly expressed and induced apoptosis by sponging miR-9 in neurons [26]. The inhibition of TUG1 inhibited the apoptosis, inflammation via harboring miR-449b-5p in renal tubular epithelial cells [38]. In accordance with previous research, the potential miRNAs (miR-493-3p and miR-410-3p) of TUG1 were predicted by online database starBase and verified by dual-luciferase reporter assay. The expression of miR-493-3p or miR-410-3p was inhibited in the OGD/R-induced cell model and the serum of CIRI patients. Besides, TUG1 acted as a molecule sponge for miR-493-3p or miR-410-3p. And inhibition of miR-493-3p or miR-410-3p restored the suppression effect on apoptosis, inflammation and oxidative stress, cytotoxicity, increase in proliferation mediated by TUG1 knockdown in OGD/R model cells, which indicated that TUG1 exerted its function in OGD/R model cells partly by regulating miR-493-3p or miR-410-3p.

MAPKs are a group of heterogeneous serine/threonine kinases that possess the ability of sensing extracellular stimulus, such as inflammatory cytokines, oxidative stress products, hormones, and regulating gene expression and cytoplasmic functions [39]. Among the MAPK system, JNK and p38 are similar in function that are associated with inflammation, apoptosis and proliferation of cells [40]. In CIRI, the downregulation of JNK and p38 signal pathways brought about the protection of neuro cells [41]. Besides, the JNK and p38 pathways were inhibited to protect the neuro cells from acrylamide-induced peripheral nervous system toxicity in rat [42]. Linc00114 sponged miR-203 to regulate JNK pathway in nasopharyngeal carcinoma [43]. In current research, anti-miR-493-3p or anti-miR-410-3p restored the inhibition effect of si-TUG1 on JNK and p38 pathways in CIRI, and these data uncovered that TUG1 activated JNK and p38 MAPK pathways by harboring miR-493-3p or miR-410-3p.

In conclusion, TUG1 was upregulated, while miR-493-3p and miR-410-3p were downregulated in the serum of CIRI patients. TUG1 activated JNK and p38 MAPK pathways by sponging miR-493-3p or miR-410-3p, thereby stimulating the apoptosis, inflammation and oxidative stress, but inhibited proliferation in OGD/R model cells. Based on these results, we concluded that TUG1 exerted as an activator in CIRI via sponging miR-493-3p or miR-410-3p and activating JNK and p38 pathways, which may provide potential clues for targeted therapy of CIRI.
